# Prevalence and Possible Risk Factors of Low Bone Mineral Density in Untreated Female Patients with Systemic Lupus Erythematosus

**DOI:** 10.1155/2015/510514

**Published:** 2015-02-11

**Authors:** Yi-Ning Sun, Xiu-Yuan Feng, Lan He, Ling-Xia Zeng, Zhi-Ming Hao, Xiao-Hong Lv, Dan Pu

**Affiliations:** ^1^Department of Rheumatology, First Affiliated Hospital of the Medical School, Xi'an Jiaotong University, Xi'an, Shaanxi 710061, China; ^2^Department of Epidemiology and Biostatistics, School of Public Health, Xi'an Jiaotong University, Xi'an, Shaanxi 710061, China

## Abstract

Systemic lupus erythematosus (SLE) is an autoimmune disease characterized by chronic inflammation. Different studies have shown decreased bone mineral density (BMD) in patients with SLE. The objective of this study was to investigate the prevalence and possible risk factors of low BMD in untreated female patients with SLE in Chinese population. A total of 119 untreated female patients with SLE were included. BMD was measured at lumbar spine and at total hip by dual-energy X-ray absorptiometry. The associations between decreased BMD and demographic variables, clinical variables, and bone metabolism variables were analyzed. These SLE patients had the following characteristics: mean age was 32.6 ± 11.9 years, mean disease duration was 22.1 ± 34.5 months, and mean SLEDAI was 11.4 ± 5.4. Osteopenia was present in 31.1% of the patients and osteoporosis in 8.5%. A significant negative association between low density lipoprotein cholesterol (LDL-c) and BMD at the lumbar spine (*correlation coefficient* = −0.242; *P* = 0.023) and total hip (*correlation coefficient* = −0.259; *P* = 0.019) was shown. These results seem to indicate that increased LDL-c may be an important risk factor for low BMD at lumbar spine and total hip in untreated female SLE patients.

## 1. Introduction

Systemic lupus erythematosus (SLE) is an autoimmune disease characterized by chronic inflammation and multisystemic damage. Studies from different parts of the world have shown osteoporosis and low bone mineral density (BMD) in patients with SLE [1, 2], especially in female patients [3]. In several cross-sectional studies, osteopenia is found in 4% to 74% of SLE patients and osteoporosis in 3% to 48% of patients with SLE [4]. The discrepancy in the prevalence between different studies may be due to different study design, sex, age, ethnicity, and disease activity, but, in general, a reduction in BMD was demonstrated in patients with SLE.

Osteoporosis is characterized by low bone mass and damaged structural integrity that lead to increased risk of bone fracture. Osteoporosis-related fragility fractures represent one of the most important complications that may occur in patients with SLE. A five-year prospective study determined the incidence of nonvertebral and vertebral fracture in Chinese SLE patients was 1.26 and 0.94 per 100 patient-years [5]. The prevalence of vertebral fracture in SLE ranges between 20% and 29% [6]. These fractures may provoke disability and contribute to an important decrease in quality of life. Moreover, long-term studies have shown vertebral fractures were associated with increased morbidity and mortality [7].

Patients with SLE are at increased risk for bone loss and fractures for many reasons. It has been established that the old age, postmenopausal status, and low body mass index are the possible risk factors for osteoporosis in SLE. Furthermore, chronic inflammation, immobility, and vitamin D deficiency due to lack of sun exposure and treatment with glucocorticoids may be common factors that substantially increase osteoporosis risk in these patients [4, 8]. The main objective of this study was therefore to investigate the incidence of osteoporosis and osteopenia in untreated female patients with SLE by using dual-energy X-ray absorptiometry (DXA) and to identify the possible risk factors associated with low BMD in the SLE patients.

## 2. Materials and Methods

### 2.1. Patients

One hundred and nineteen untreated female patients with SLE were included in this study from August 2012 to August 2014. All patients attended the rheumatology clinic of the First Affiliated Hospital of the Medical School, Xi'an Jiaotong University. All patients fulfilled the 1997 American College of Rheumatology (ACR) revised criteria for the classification of SLE [9] and were provided written consent for participation. This study was approved by the Ethics Committee of the hospital.

### 2.2. BMD Measurements

BMD at the lumbar spine (anteroposterior, L1–L4) and total hip (left femoral neck) were performed by a single trained technician using dual-energy X-ray absorptiometry (LEXXOS-LX381, French DMS). All measurements were performed in accordance with standard instrument procedures and matched gender and weight. The precisions for duplicate measurements were 0.16% for lumbar spine and 0.15% for total hip. All BMD results were expressed in absolute values (g/cm^2^) and as the number of standard deviations (SD) above or below the mean results of young female adults, *T* score, and compared with an age matched female reference population, *Z* score. In this study, osteoporosis was defined as a *T* score or *Z* score ≤−2.5 in lumbar spine or total hip and osteopenia as a *T* score or *Z* score <−1 but >−2.5.

### 2.3. Clinical Data Collection and Analysis

Demographic data collected at the time of study inclusion were age, body weight, height, body mass index (BMI), menstrual status, and age at menopause. In addition, we inquired about lifestyle related data including smoking, intake of alcohol, history of lumbar or femoral head fracture, and family history of osteoporosis and fracture.

Clinical data of SLE patients were assessed for disease duration, serum albumin, serum creatinine, 24-hour urinary protein, serum levels of total cholesterol (TC), triglyceride, low density lipoprotein cholesterol (LDL-c), erythrocyte sedimentation rate (ESR), and C-reactive protein (CRP). Immunologic measures were regarding complement components and anti-double stranded DNA antibodies (anti-dsDNA antibodies). Disease activity was scored using the Systemic Lupus Erythematosus Disease Activity Index (SLEDAI) by the physicians in charge of the patients [10].

Biochemical and hormonal variables related to bone mineral metabolism such as serum levels of calcium, phosphate, and parathyroid hormone (PTH), levels of 25-hydroxyvitamin D (25[OH]D), bone formation marker (Osteocalcin, OC), and bone resorption marker (C-terminal cross-linking telopeptide of type I collagen, CTX) were examined. All venous blood samples were taken after a one-night fast and analyzed consecutively using standard laboratory techniques in the Department of Clinical Laboratory at our hospital. Based on the laboratory reference value, deficiency of 25(OH)D was defined as a serum level <20 ng/milliliter. In female, a high titer of CTX was defined as a serum level >573 pg/milliliter during premenopause and >1008 pg/milliliter during postmenopause.

### 2.4. Statistical Analysis

SPSS 16.0 for Windows statistical software was used for data analysis. Measurement data were presented as the mean ± standard deviation (mean ± SD). The Student *t*-test and Mann-Whitney *U* test were used to analyze parametric and nonparametric variables. Variables possibly associated with a decreased BMD at the lumbar spine and at the hip were examined by Pearson's correlation coefficients or Spearman's correlation coefficients. The following variables were examined in relationship to BMD: age, BMI, disease duration, disease activity, serum albumin, serum creatinine, 24-hour urine protein, serum TC, triglyceride and LDL-c, ESR, CRP, complement components, anti-dsDNA antibodies, serum calcium, phosphate, PTH, serum 25[OH]D, OC, and CTX. A *P* value less than or equal to 0.05 (2-sided) was considered statistically significant.

## 3. Results

### 3.1. Demographic, Clinical, and Bone Metabolism Variables in SLE Patients

One hundred and nineteen untreated female SLE patients were included in the study. The demographic, clinical, and bone metabolism characteristics of the patients are shown in Table 1. The median age of the participants was 32.6 ± 11.9 (10~67) yrs. Fourteen patients (11.9%) were postmenopausal and the median age at menopause was 48.2 ± 1.9 (45~51) yrs. The mean body mass index (BMI) of the patients was 20.6 ± 3.3 kg/m^2^. Mean disease duration was 22.1 ± 34.5 months and mean SLEDAI was 11.4 ± 5.4.

### 3.2. BMD in SLE Patients

The results of the BMD measurements are shown in Table 2. The mean BMD ± SD was 0.91 ± 0.16 g/cm^2^ at the lumbar spine and 0.88 ± 0.12 g/cm^2^ at total hip. The mean *T* score ± SD was −0.45 ± 1.40 at the lumbar spine and −0.01 ± 1.08 at total hip. The frequency of osteopenia (*T* score or *Z* score less than −1.0 SD at the lumbar spine [L1–L4] and/or at the total hip) was 31.1%. The frequency of osteoporosis (*T* score or *Z* score less than −2.5 SD at the lumbar spine [L1–L4] and/or at the total hip) was 8.5%.

### 3.3. Characteristics of Patients with Low BMD

We further analyzed and compared the SLE patients with normal or low BMD at the lumbar spine or at total hip, as shown in Table 3. Serum total cholesterol was significantly increased in patients with low BMD (4.2 ± 1.8 mmol/liter) than those in normal BMD (3.3 ± 0.9 mmol/liter, *P* = 0.007). Serum low density lipoprotein cholesterol was also increased in patients with low BMD (2.4 ± 1.2 mmol/liter) than those in normal BMD (1.9 ± 0.6 mmol/liter). However, the difference was not statistically significant (*P* = 0.055).

### 3.4. Variables Associated with Low Bone Mineral Density

To reveal the risk factors that may relate to the low BMD, we analyzed the demographic, clinical, and bone metabolism variables in patients with abnormal BMD as shown in Table 4. We found that serum low density lipoprotein cholesterol correlated negatively with low BMD at the lumbar spine (correlation coefficient = −0.242, *P* = 0.023) and at the hip significantly (correlation coefficient = −0.259, *P* = 0.019). However, we found no significant correlation between serum total cholesterol and low BMD at lumbar spine and at total hip. BMD at the lumbar spine and the hip was not associated with age, BMI, disease duration, disease activity, ESR, CRP, and complement components. Serum 25[OH]D was considered and analyzed as a potential risk factor for reduced BMD, but no statistically significant correlation was found.

## 4. Discussion

In patients with systemic lupus erythematosus (SLE), an increase in bone loss and fragility fracture was found compared with general population [11]. Recent studies have shown a prevalence of osteopenia of 28–46% and a prevalence of osteoporosis of 3–9% among SLE patients [12, 13]. The discrepancy among the different studies may be due to sex, ages, menopausal status, ethnicity, different disease duration, and different disease activity. For example, BMD in postmenopausal patients with SLE (osteopenia 74%; osteoporosis 48%) was more dramatically decreased than that in premenopausal patients (osteopenia 40%; osteoporosis 5%) [2, 14]. Racial difference in the prevalence of low BMD in SLE patients may exist. Lee et al. [15] found that African American women had lower BMD at the hip and lumbar spine compared with white women with SLE. Three studies reported the prevalence of osteopenia in 30% to 50% and osteoporosis in 13.8% to 48% of Asian female SLE patients [1, 14, 16]. We demonstrated a prevalence of osteopenia of 31.1% and a prevalence of osteoporosis of 8.5% among untreated female SLE patients. Our findings were in consistency with previous studies of SLE patients, showing reduced bone mass at lumbar spine and at the hip in SLE patients.

Interestingly, the higher proportion of osteopenia and osteoporosis at the lumbar spine (25.5%; 10.4%) when compared with the hip (7.5%; 0.9%) was observed in our study (Table 2). The reasons for this discrepancy are unclear but may to some extent be related to variations in the type of bone comprising these two anatomic sites [17]. Glucocorticoids may be considered an important factor to induce bone loss in SLE patients, as glucocorticoids can directly inhibit osteoblasts and thus reduce the bone formation [18]. The lumbar spine, which is composed predominantly of trabecular-rich bone, is more susceptible to the adverse effects of corticosteroids than the hip that consists of comparatively less trabecular bone. In the present study, untreated SLE patients were included in order to exclude glucocorticoids exposure which may influence BMD measurements. In fact, studies have observed trabecular bone loss in patients with SLE that could not be explained by corticosteroids use [19]. Apart from glucocorticoids, a rapid loss of spine trabecular bone in untreated SLE patients is likely to arise from chronic inflammation and disease damage [20] and several studies have shown the high prevalence of vertebral fractures in SLE patients [6, 21–23]. Cross-sectional studies have found that low BMD at the hip remained as a significant factor for the presence of vertebral fracture [6, 22]. In fact, osteoporosis at the lumbar spine may be one of the contribution factors associated with both new vertebral and nonvertebral fractures [5].

Various risk factors and clinical variables have been demonstrated to associate with decreased BMD in the spine and hip of SLE patients [4, 8]. In our study of untreated female SLE patients, we found a significant negative correlation between low BMD at the lumbar spine (correlation coefficient = −0.242, *P* = 0.023) and at total hip (correlation coefficient = −0.259, *P* = 0.019) and serum LDL-c, indicating the importance of increased LDL-c as a major risk factor for low BMD at lumbar spine and at total hip. We could not find the correlation between BMI, disease activity, 25(OH)D, ESR, CRP, complement components, and low BMD at the lumbar spine and at the hip. These findings were consistent with two large-population studies in Indian and Korean population, which identified that LDL-c and TC had weak but significant negative correlation with BMD at lumbar spine and at the hip in men and pre- and postmenopausal women [23, 24]. A lower fat intake and body fatness as well as a better profile of LDL-c predicted high BMD in healthy women [25].

Although atherosclerosis and osteoporosis are considered to be different in pathology, they share bidirectional correlation [26]. On the one hand, low bone density is associated with incident cardiovascular disease [27]. On the other hand, women with cardiovascular disease have increased risk of osteoporotic fracture [28]. In patients with SLE, decreased BMD is an independent predictor for premature coronary calcification [29]. The definite link between atherosclerosis and osteoporosis suggested that they may share common risk factors. Oxidized LDL is well known to play an important role in the generation and progression of atherosclerosis. So it may be considered as a candidate that links both disorders. In fact, it has been shown that high serum LDL-c level may be a risk factor for low BMD [23] and for nonvertebral fragility fracture [30]. Increased level of oxidized LDL-c was demonstrated in patients with active lupus and chronic systemic inflammation including dyslipidemia may contribute to the bone loss in SLE [31]. Experiments in mice have shown that oxidized lipid may attenuate osteogenesis and parathyroid hormone bone anabolism is blunted in hyperlipidemic mice [32]. Oxidized LDL may negatively influence bone formation by reducing osteoblast maturation and phosphate-induced mineralization in osteoblasts [33]. Oxidized LDL increases the expression of receptor activator of nuclear factor kappa-B ligand (RANKL) on osteoblast via extracellular signal regulated kinase (ERK), nuclear factor *κ*B (NF*κ*B), and nuclear factor of activated-T-cell (NFAT) signaling pathways [34]. RANKL can stimulate osteoclast survival, activation, and differentiation, so it is conceivable to infer that increased level of RANKL may cause a decrease in BMD. In addition, since RANKL is involved in myocardial inflammation, vascular calcification, and plaque rupture, the increase in RANKL in prooxidant conditions observed in endothelial cells and fibroblasts may link the atherosclerosis to osteoporosis [35].

In conclusion, we measured the BMD at lumbar spine and at total hip of 119 untreated female patients with SLE. The incidences of osteopenia and osteoporosis in these patients were 31.1% and 8.5%, respectively. We found that the patients with low BMD had increased LDL-c level in the serum and that increased LDL-c correlates negatively with low BMD at the lumbar spine and at total hip. Our results reveal a new risk factor for the bone loss in SLE and may shed some light on the future prevention and treatment of osteopenia and osteoporosis in female SLE patients.

## Figures and Tables

**Table 1:** Demographic, clinical data and bone metabolic variables in the SLE patients.

Variable	All SLE patients (n = 119)
Demographic variables	
Age, mean ± SD years	32.6 ± 11.9
Body mass index, mean ± SD kg/m^2^	20.6 ± 3.3
Postmenopausal, %	11.9
Menopausal age, mean ± SD years	48.2 ± 1.9
Clinical variables	
Disease duration, mean ± SD months	22.1 ± 34.5
Serum creatinine, mean ± SD *μ*mol/liter	53.9 ± 23.3
Serum albumin, mean ± SD g/liter	31.7 ± 6.9
24-hour urine protein, mean ± SD g/24 hr	0.58 ± 0.84
Serum total cholesterol, mean ± SD mmol/liter	3.7 ± 1.4
Serum triglyceride, mean ± SD mmol/liter	2.0 ± 1.3
Serum low density lipoprotein cholesterol, mean ± SD mmol/liter	2.1 ± 0.9
Erythrocyte sedimentation rate, mean ± SD mm/hour	53.8 ± 34.7
C-reactive protein, mean ± SD mg/liter	11.1 ± 13.4
Complement 3, mean ± SD g/liter	0.57 ± 0.31
Complement 4, mean ± SD g/liter	0.10 ± 0.09
Anti-dsDNA antibodies, mean ± SD, IU/milliliter	43.7 ± 37.9
SLEDAI, mean ± SD	11.4 ± 5.4
Bone metabolism variables	
25-Hydroxyvitamin D, mean ± SD ng/milliliter	11.9 ± 7.3
Serum calcium, mean ± SD mmol/liter	2.0 ± 0.2
Serum phosphate, mean ± SD mmol/liter	1.1 ± 0.3
Parathyroid hormone (PTH), mean ± SD pg/milliliter	36.2 ± 22.9
Osteocalcin (OC), mean ± SD ng/milliliter	10.3 ± 6.7
C-terminal cross-linking telopeptide of type I collagen (CTX), mean ± SD pg/milliliter	557.3 ± 396.2

SLE = systemic lupus erythematosus; SLEDAI = Systemic Lupus Erythematosus Disease Activity Index (range 0–105).

**Table 2:** Lumbar spine and total hip BMD in the study of SLE patients.

Variable	All SLE patients (*n* = 119)
BMD, mean ± SD g/cm^2^	
Spine L1–L4	0.91 ± 0.16
Total hip	0.88 ± 0.12
*T* score, mean ± SD	
Spine L1–L4	−0.45 ± 1.40
Total hip	−0.01 ± 1.08
*Z* score, mean ± SD	
Spine L1–L4	−0.36 ± 1.34
Total hip	0.62 ± 1.03
Osteopenia, %	
Lumbar spine or/and total hip	31.1
Lumbar spine	25.5
Total hip	10.4
Osteoporosis, %	
Lumbar spine or/and total hip	8.5
Lumbar spine	7.5
Total hip	0.9

SLE = systemic lupus erythematosus; BMD = bone mineral density.

**Table 3:** Characteristics of SLE patients with normal and low BMD at lumbar spine and total hip.

Variable	Normal BMD	Low BMD	*P*
Demographic variables			
Age^#^	31.6 ± 8.9	33.9 ± 16.1	0.869
BMI^#^	20.7 ± 3.5	20.3 ± 2.8	0.730
Clinical variables			
Disease duration^#^	24.1 ± 34.7	20.1 ± 34.5	0.895
Serum creatinine^#^	54.6 ± 26.7	55.2 ± 20.3	0.651
Serum albumin^#^	31.7 ± 5.8	32.3 ± 8.6	0.216
24-hour urine protein^#^	0.56 ± 0.75	0.62 ± 1.02	0.586
Serum TC^#^	3.3 ± 0.9	4.2 ± 1.8	0.007
Serum triglyceride^#^	2.0 ± 1.1	2.0 ± 1.7	0.332
Serum LDL-c^#^	1.9 ± 0.6	2.4 ± 1.2	0.055
ESR^#^	56.1 ± 34.7	54.9 ± 33.0	0.948
CRP^#^	10.8 ± 12.9	10.8 ± 12.7	0.963
Complement 3^#^	0.57 ± 0.32	0.59 ± 0.33	0.892
Complement 4^#^	0.10 ± 0.09	0.10 ± 0.09	0.909
SLEDAI^#^	11.7 ± 6.0	10.8 ± 5.2	0.579
Bone metabolism variables			
Serum 25-[OH]D^#^	12.2 ± 7.8	11.8 ± 6.7	0.995
Serum calcium^*^	2.0 ± 0.2	2.0 ± 0.2	0.978
Serum phosphate^*^	1.2 ± 0.3	1.0 ± 0.3	0.715
PTH^#^	34.8 ± 23.7	38.1 ± 21.3	0.342
OC^#^	10.1 ± 5.5	11.2 ± 7.9	0.880
CTX^#^	500.8 ± 357.8	633.7 ± 498.0	0.373

^*^The Student *t*-test; ^#^Mann-Whitney *U* test.

BMD = bone mineral density; BMI = body mass index; TC = total cholesterol; LDL-c = low density lipoprotein cholesterol; ESR = erythrocyte sedimentation rate; CRP = C-reactive protein; SLEDAI = Systemic Lupus Erythematosus Disease Activity Index; 25-[OH]D = 25-hydroxyvitamin D; PTH = parathyroid hormone; OC = osteocalcin; CTX = C-terminal cross-linking telopeptide of type I collagen.

**Table 4:** Relationship between BMD at lumbar spine and total hip and demographic, clinical, and bone metabolism variables by univariate analysis.

Variable	BMD in lumbar spine		BMD in total hip	
	Correlation coefficient	P	Correlation coefficient	P
Demographic variables				
Age^#^	0.028	0.776	−0.153	0.136
BMI^*^	0.051	0.603	0.160	0.119
Clinical variables				
Disease duration^#^	−0.036	0.717	−0.077	0.457
Serum albumin^#^	−0.166	0.091	−0.088	0.395
Serum creatinine^#^	−0.041	0.677	−0.167	0.104
24-hour urine protein^#^	−0.020	0.856	−0.068	0.561
Serum TC^#^	−0.193	0.052	−0.135	0.198
Serum triglyceride^#^	0.129	0.228	−0.009	0.936
Serum LDL-c^*^	−0.242	0.023	−0.259	0.019
ESR^#^	−0.084	0.401	−0.013	0.905
CRP^#^	−0.089	0.401	−0.085	0.439
Complement 3^#^	−0.084	0.395	0.033	0.751
Complement 4^#^	−0.068	0.493	−0.095	0.361
SLEDAI^#^	0.012	0.907	0.109	0.290
Bone metabolism variables				
Serum 25-[OH]D^#^	−0.024	0.810	0.094	0.371
Serum calcium^*^	−0.125	0.215	0.094	0.373
Serum phosphate^*^	0.130	0.216	0.187	0.089
PTH^#^	−0.147	0.154	−0.162	0.128
OC^#^	−0.055	0.588	0.011	0.916
CTX^#^	−0.152	0.170	−0.163	0.164

^*^Pearson's correlation coefficients; ^#^Spearman's correlation coefficients.

BMD = bone mineral density; BMI = body mass index; TC = total cholesterol; LDL-c = low density lipoprotein cholesterol; ESR = erythrocyte sedimentation rate; CRP = C-reactive protein; SLEDAI = Systemic Lupus Erythematosus Disease Activity Index; 25-[OH]D = 25-hydroxyvitamin D; PTH = parathyroid hormone; OC = osteocalcin; CTX = C-terminal cross-linking telopeptide of type I collagen.
